# Genetically predicted telomere length and Alzheimer’s disease endophenotypes: a Mendelian randomization study

**DOI:** 10.1186/s13195-022-01101-9

**Published:** 2022-11-07

**Authors:** Blanca Rodríguez-Fernández, Natalia Vilor-Tejedor, Eider M. Arenaza-Urquijo, Gonzalo Sánchez-Benavides, Marc Suárez-Calvet, Grégory Operto, Carolina Minguillón, Karine Fauria, Gwendlyn Kollmorgen, Ivonne Suridjan, Manuel Castro de Moura, David Piñeyro, Manel Esteller, Kaj Blennow, Henrik Zetterberg, Immaculata De Vivo, José Luis Molinuevo, Arcadi Navarro, Juan Domingo Gispert, Aleix Sala-Vila, Marta Crous-Bou, for the ALFA study, Müge Akinci, Annabella Beteta, Anna Brugulat-Serrat, Raffaele Cacciaglia, Alba Cañas, Irene Cumplido, Carme Deulofeu, Ruth Dominguez, Maria Emilio, Carles Falcon, Sherezade Fuentes, Oriol Grau-Rivera, José M. González-de-Echávarri, Laura Hernandez, Patricia Genius, Gema Huesa, Jordi Huguet, Eva M. Palacios, Paula Marne, Tania Menchón, Marta Milà-Alomà, Cleofé Peña-Gomez, Albina Polo, Sandra Pradas, Gemma Salvadó, Mahnaz Shekari, Anna Soteras, Laura Stankeviciute, Marc Vilanova

**Affiliations:** 1 BarcelonaBeta Brain Research Center (BBRC), Pasqual Maragall Foundation, C. Wellington 30, Barcelona, 08005 Spain; 2 Centre for Genomic Regulation (CRG), The Barcelona Institute for Science and Technology, Barcelona, Spain; 3 Department of Clinical Genetics, Erasmus University Medical Center, Rotterdam, the Netherlands; 4 Universitat Pompeu Fabra (UPF), Barcelona, Spain; 5 IMIM - Hospital del Mar Medical Research Institute, Barcelona, Spain; 6 Centro de Investigación Biomédica en Red de Fragilidad y Envejecimiento Saludable (CIBER-FES), Instituto de Salud Carlos III, Madrid, Spain; 7 Servei de Neurologia, Hospital del Mar, Barcelona, Spain; 8 Roche Diagnostics GmbH, Penzberg, Germany; 9 Roche Diagnostics International Ltd, Rotkreuz, Switzerland; 10 Josep Carreras Leukaemia Research Institute (IJC), Badalona, Barcelona, Spain; 11 Centro de Investigación Biomédica en Red Cáncer (CIBERONC), Madrid, Spain; 12 Institució Catalana de Recerca i Estudis Avançats (ICREA), Barcelona, Catalonia Spain; 13 Physiological Sciences Department, School of Medicine and Health Sciences, University of Barcelona (UB), Barcelona, Catalonia Spain; 14 Department of Psychiatry and Neurochemistry, Institute of Neuroscience and Physiology, University of Gothenburg, Mölndal, Sweden; 15 Clinical Neurochemistry Laboratory, Sahlgrenska University Hospital, Mölndal, Sweden; 16 UK Dementia Research Institute at UCL, London, UK; 17 Department of Neurodegenerative Disease, UCL Institute of Neurology, Queen Square, London, UK; 18 Hong Kong Center for Neurodegenerative Diseases, Hong Kong, China; 19 Department of Epidemiology, Harvard T.H. Chan School of Public Health, Boston, MA USA; 20 Channing Division of Network Medicine, Harvard Medical School, Boston, MA USA; 21 H. Lundbeck A/S, Copenhagen, Denmark; 22 Institute of Evolutionary Biology (CSIC-UPF), Department of Experimental and Health Sciences, Universitat Pompeu Fabra, Barcelona, Spain; 23 Centro de Investigación Biomédica en Red Bioingeniería, Biomateriales y Nanomedicina, Madrid, Spain; 24 Centro Nacional de Investigaciones Cardiovasculares (CNIC), Madrid, Spain; 25 Unit of Nutrition and Cancer, Cancer Epidemiology Research Program, Catalan Institute of Oncology (ICO)-Bellvitge Biomedical Research Center (IDIBELL), Avda. Gran Via de l’Hospitalet 199-203, 08908 l’Hospitalet de Llobregat, Barcelona, Spain

**Keywords:** Alzheimer’s disease, Cerebrospinal fluid biomarkers, Mendelian randomization, Neuroimaging, Polygenic risk score, Telomere length

## Abstract

Telomere length (TL) is associated with biological aging, consequently influencing the risk of age-related diseases such as Alzheimer’s disease (AD). We aimed to evaluate the potential causal role of TL in AD endophenotypes (i.e*.*, cognitive performance, *N* = 2233; brain age and AD-related signatures, *N* = 1134; and cerebrospinal fluid biomarkers (CSF) of AD and neurodegeneration, *N* = 304) through a Mendelian randomization (MR) analysis. Our analysis was conducted in the context of the ALFA (ALzheimer and FAmilies) study, a population of cognitively healthy individuals at risk of AD. A total of 20 single nucleotide polymorphisms associated with TL were used to determine the effect of TL on AD endophenotypes. Analyses were adjusted by age, sex, and years of education. Stratified analyses by *APOE*-ɛ4 status and polygenic risk score of AD were conducted. MR analysis revealed significant associations between genetically predicted longer TL and lower levels of CSF Aβ and higher levels of CSF NfL only in *APOE*-ɛ4 non-carriers. Moreover, inheriting longer TL was associated with greater cortical thickness in age and AD-related brain signatures and lower levels of CSF p-tau among individuals at a high genetic predisposition to AD. Further observational analyses are warranted to better understand these associations.

**Graphical Abstract:**

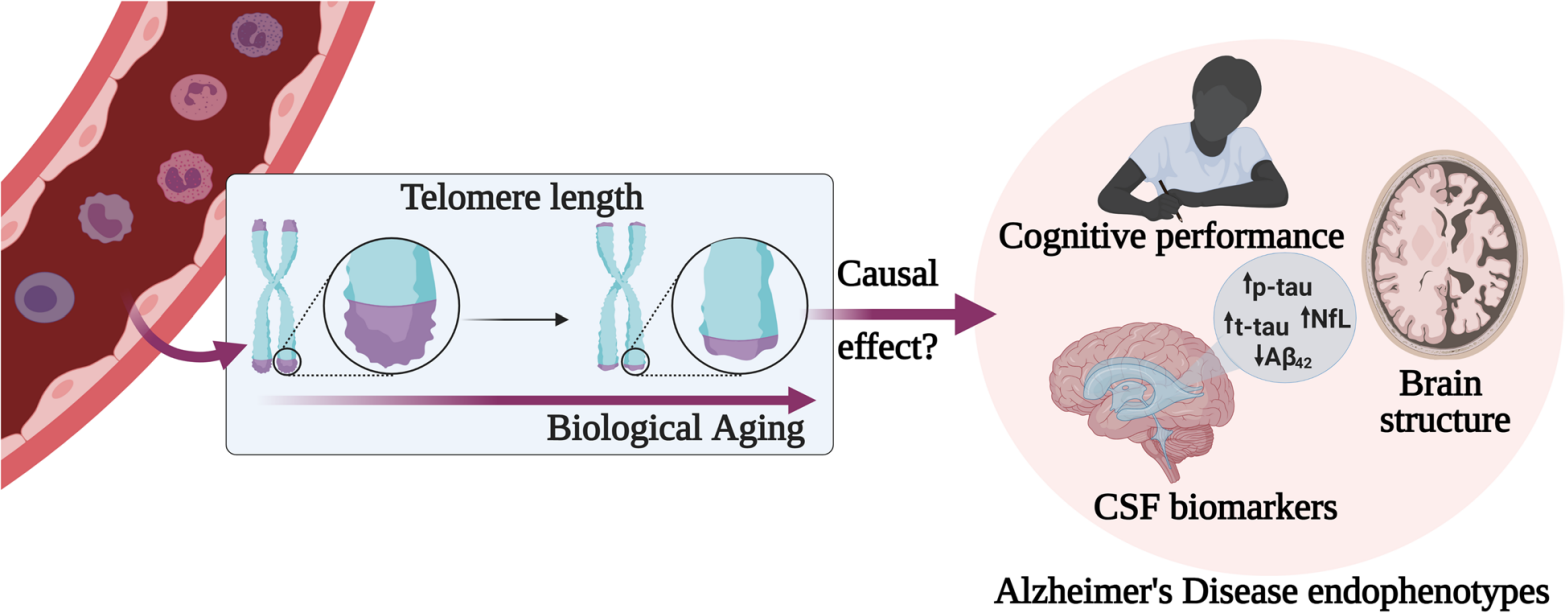

**Supplementary Information:**

The online version contains supplementary material available at 10.1186/s13195-022-01101-9.

## Introduction 

Telomere length (TL) is a candidate biomarker of biological aging and aging-related outcomes [1, 2]. Telomeres are tandem deoxyribonucleic acid (DNA) repeats of a short sequence unit located at the end of the eukaryotic linear chromosomes that undergo attrition every time a somatic cell divides [3]. Shorter telomeres are associated with mortality [4], increased rates of age-related diseases [5–8], progression to dementia [9], and higher overall risk of developing Alzheimer’s disease (AD) [10, 11].

Previous studies have reported associations between TL and cognitive performance [12] as well as cognitive decline in cognitively unimpaired individuals [13]. Furthermore, TL is thought to affect brain structure; shorter TL has been associated with cortical atrophy in healthy subjects [14]. However, both longer and shorter TL have been associated with lower hippocampal volumes in the general population [15–19]. To date, no studies have evaluated the impact of TL in cortical or hippocampus regions differentially affected by AD or aging-related processes. Moreover, the potential interplay between TL and AD pathology remains to be elucidated. TL has been shown to be independent of amyloid-β (Aβ) deposition in AD patients [20, 21]. In contrast, Mahoney et al. [22] described an interaction between longer TL, cognitive decline, and abnormal levels of cerebrospinal fluid (CSF) Aβ and tau in participants of the AD Neuroimaging Initiative.

TL and AD are affected by common lifestyle-related risk factors [23, 24]. Among other reasons, these common factors may be confounders and lead to reverse causation in observational studies, partially explaining the heterogeneity of results found in the literature. The Mendelian randomization (MR) approach was developed to overcome these limitations. Unlike observational studies, the MR approach does not explore the association directly but through genetic instrumental variables (e.g., single nucleotide polymorphisms, SNPs) whose estimation of TL is not influenced by confounding or reverse causation [25, 26]. Previous MR studies have described potential causal effects of genetically predicted TL on the risk of AD [27–30]. However, other MR studies showed inconsistent results regarding TL and cognitive traits [31, 32].

The aim of the present study was to evaluate whether TL may play a causal role in the underlying pathophysiological mechanisms of AD by influencing its endophenotypes measured as cognitive performance, brain structure, and CSF biomarkers of AD and neurodegeneration, in a sample of cognitively unimpaired individuals at increased risk of AD.

## Methods

### Study participants

The present is a cross-sectional analysis that was conducted in the context of an existing study, the ALFA (ALzheimer and FAmilies) study [33]. Briefly, the ALFA study (Clinicaltrials.gov Identifier: NCT01835717) includes a total of 2573 cognitively unimpaired participants, aged 45–74, many of them kindred of AD patients (86% had at least one parent with dementia regardless of age at onset, 48% of the participants had at least one parent diagnosed with AD before the age of 75).

Participants were characterized at their baseline visit in 2013–2014 at multiple levels (sociodemographic, anthropometric, clinical, epidemiological, cognitive, and neuroimaging). For a subset of the ALFA study participants, we additionally collected blood samples for further genetic analysis. Moreover, a subset of the ALFA study participants (ALFA+ study) underwent lumbar puncture for CSF biomarker analysis. ALFA exclusion criteria were [1] cognitive performance falling outside established cutoffs: Mini-Mental State Examination [34, 35] < 26, Memory Impairment Screen [36, 37] < 6, Time-Orientation subtest of the Barcelona Test II [38] < 68, semantic fluency [39, 40] (animals) < 12, and Clinical Dementia Rating scale [41] > 0; [2] major psychiatric disorders (DSM-IV-TR) or diseases that could affect cognitive abilities; [3] severe auditory and/or visual, neurodevelopmental, and/or psychomotor disorders, significant diseases that could interfere with cognition; [4] neurological disorders; [5] brain injury that could interfere with cognition; and [6] family history of AD with a suspected autosomal dominant pattern. Further details of ALFA participants can be found in Molinuevo et al. [33].

The study was conducted in accordance with the directives of the Spanish Law 14/2007, of 3rd of July, on Biomedical Research (Ley 14/2007 de Investigación Biomédica). All participants accepted the study procedures by signing an informed consent form.

### Genetic data 

#### Genotyping

DNA samples were obtained from whole blood samples by applying a salting-out protocol. DNA was eluted in 800μl of H_2_O (Milli-Q) and quantified using Quant-iTT PicoGreen® dsDNA Assay Kit (Life Technologies) at the Cancer Epigenetics and Biology Program (PEBC; IDIBELL, Barcelona, Spain). The DNA concentration for each sample was additionally normalized. Genome-wide genotyping was performed using the Illumina Infinim NeuroChip [42] backbone version 1.0 and 1.2, based on the genome-wide genotyping array (Infinium HumanCore-24 v1.0). PLINK was used for the quality control (QC) of the genetic data [43]. We applied the following sample QC thresholds: sample missingness rates > 2% and heterozygosity 3 standard deviations (SD). Individuals showing sex discordances and high percentage of shared alleles (i.e., identity-by-descent (IBD) > 0.185) [44] were also excluded. A total of 2280 individuals remain after the genetic QC procedure.

#### Imputation

Genetic imputation was done using the Michigan imputation server, according to established guidelines (https://imputationserver.sph.umich.edu) and the European ancestry haplotype reference panel (HRC r1.1 2016) [45, 46]. The genetic datasets were phased using EAGLE software v.2.4, and data were rechecked using the default QC in the Michigan imputation server. After imputation, 15,805,054 variants were obtained with an imputation quality of 90% (*r*-squared parameter > 0.1).

#### Genetic variants associated with longer telomere length

From the final genetic sample of the ALFA study, 20 SNPs independently associated with mean TL (*p*-val < 5 × 10^−8^) in a genome-wide meta-analysis of 78,592 individuals were selected [47]. Proxies for those SNPs were included through LDlink [48] when the genetic information was not available in our sample. Proxies were considered as those SNPs with *r*^2^ ≥ 0.9, except for the genetic variants rs55749605 (*r*^2^ = 0.84) and rs7705526 (*r*^2^ = 0.79). See the list of *r*^2^ in Additional file 1: Supplementary Table 1. Departures from the Hardy-Weinberg equilibrium and allele frequencies were inspected using the *compareGroups R* package [49]. Effect sizes (i.e., SD change in TL per copy of the effect allele) and standard errors (SE) were obtained from summary statistics from Li et al. [47]. Detailed information of those SNPs is presented in Additional file 1: Supplementary Table 1.

#### Apolipoprotein (APOE) genotyping

The *APOE* allelic variants were obtained from allelic combinations of the rs429358 and rs7412 polymorphisms [50]. According to the genotypes of these polymorphisms, subjects were classified depending on the status of the ε4 allele (non-carriers, carriers of at least one ε4 allele). For the purpose of the present study, *APOE-*ε2/ε4 individuals (*N* = 47) were excluded.

#### Polygenic risk score of Alzheimer’s disease

Genetic variants that passed the post-imputation QC were used to compute the polygenic risk score (PRS) for AD (PRS-AD) using PRSice version 2 [51]. PRSice combined AD GWAS hits (*p*-value < 5 × 10^−8^), including the *APOE* region, by summing up all the SNP alleles carried by the participants weighted by the SNP allele effect size estimated in a previous GWAS [52]. PRS-AD was Z-standardized. Individuals with a PRS-AD above percentile 75th were classified within the high genetic predisposition group, whereas those with lower PRS were classified within the low genetic predisposition group.

### Cognitive performance evaluation

During a neuropsychological evaluation at baseline, all participants were administered a cognitive test battery for the detection of early decline in longitudinal follow-up. This battery assesses episodic verbal memory measured by means of the Memory Binding Test (MBT) [53, 54], as well as executive and reasoning functions assessed by the Wechsler Adult Intelligence Scale (WAIS)-IV including psychomotor speed, visual processing, executive function (EF), and non-verbal and verbal reasoning (coding, visual puzzles, digit span, matrix reasoning, and similarities) [55]. A composite to assess cognitive performance was created based on the Preclinical Alzheimer Cognitive Composite (PACC) [56]. For our study, a modified-PACC (mPACC) composite was created by averaging the *Z*-scores of the following variables: MBT immediate total paired recall, MBT delayed free recall, WAIS-IV coding, and semantic fluency. Moreover, two additional cognitive composites to assess global episodic memory (EM) and EF were calculated by creating *Z*-scores for the cognitive measures from MBT and from WAIS-IV subtests, respectively. These global measures were calculated by averaging normalized raw scores of all subtests in each domain [57]. As with the individual cognitive tests, higher scores in the different composites represent better cognitive performance, while lower scores correspond to worse cognitive performance. EM, EF, and mPACC were used as the main outcomes to test the cross-sectional association between genetically predicted TL and cognitive performance. Data on cognitive performance was available for a total of 2233 participants.

### Neuroimaging features: image acquisition and signature calculation

The acquisition of neuroimaging data was performed for a subset of our participants through magnetic resonance imaging (MRI). MRI scans were obtained with a 3-T scanner (Ingenia CX, Philips, Amsterdam, Netherlands). The MRI protocol was identical for all participants and included a high-resolution 3D T1-weighted turbo field echo (TFE) sequence (voxel size 0.75 × 0.75 × 0.75 mm, TR/TE: 9.90/4.6 ms, flip angle = 8°). Structural T1-weighted images were segmented using FreeSurfer version 6.0 [58]. The average of the cortical thickness between hemispheres of specific brain regions was used to calculate the AD and aging brain signatures. AD brain signature was calculated as the average of the cortical thickness of specific regions known to be affected in AD (i.e., AD vulnerable brain regions): medial temporal, inferior temporal, temporal pole, superior parietal, precuneus, angular, supramarginal, superior frontal, and middle frontal [59]. Aging brain signature was calculated as the average of the cortical thickness of specific brain regions known to be affected by aging: calcarine, caudal insula, cuneus, caudal fusiform, dorsomedial frontal, lateral occipital, precentral, and inferior frontal [60]. We used AD and aging brain signatures as the main outcomes to test the association between genetically predicted TL and brain structure. Higher values in these signatures represent a thicker cortex of the areas included in the signature. AD and aging brain signatures values were available for a total of 1134 participants.

### CSF biomarkers: collection and measurement

CSF biomarkers were acquired from lumbar punctures for a subset of participants. In brief, CSF total-tau (t-tau) and phosphorylated-tau (p-tau) were measured using the electrochemiluminescence immunoassays Elecsys® phospho-tau(181P) CSF and total-tau CSF, respectively, on a fully automated cobas e 601 instrument (Roche Diagnostics International Ltd.). The rest of the CSF biomarkers Aβ42, Aβ40 and neurofilament light (NfL), were measured with in vitro diagnostic assays and robust prototype assays as part of the NeuroToolKit (Roche Diagnostics International Ltd, Rotkreuz, Switzerland) on both cobas e 601 and e 411 instruments. All measurements were performed at the Clinical Neurochemistry Laboratory, Sahlgrenska University Hospital, Mölndal, Sweden.

The CSF Aβ_42/40_ ratio was used as a biomarker of Aβ pathology [61]. The aforementioned biomarkers were used as the main outcomes to test the cross-sectional association between genetically predicted TL and core AD and neurodegeneration biomarkers in a total of 304 participants.

### Statistical analysis

**Fig. 1:**
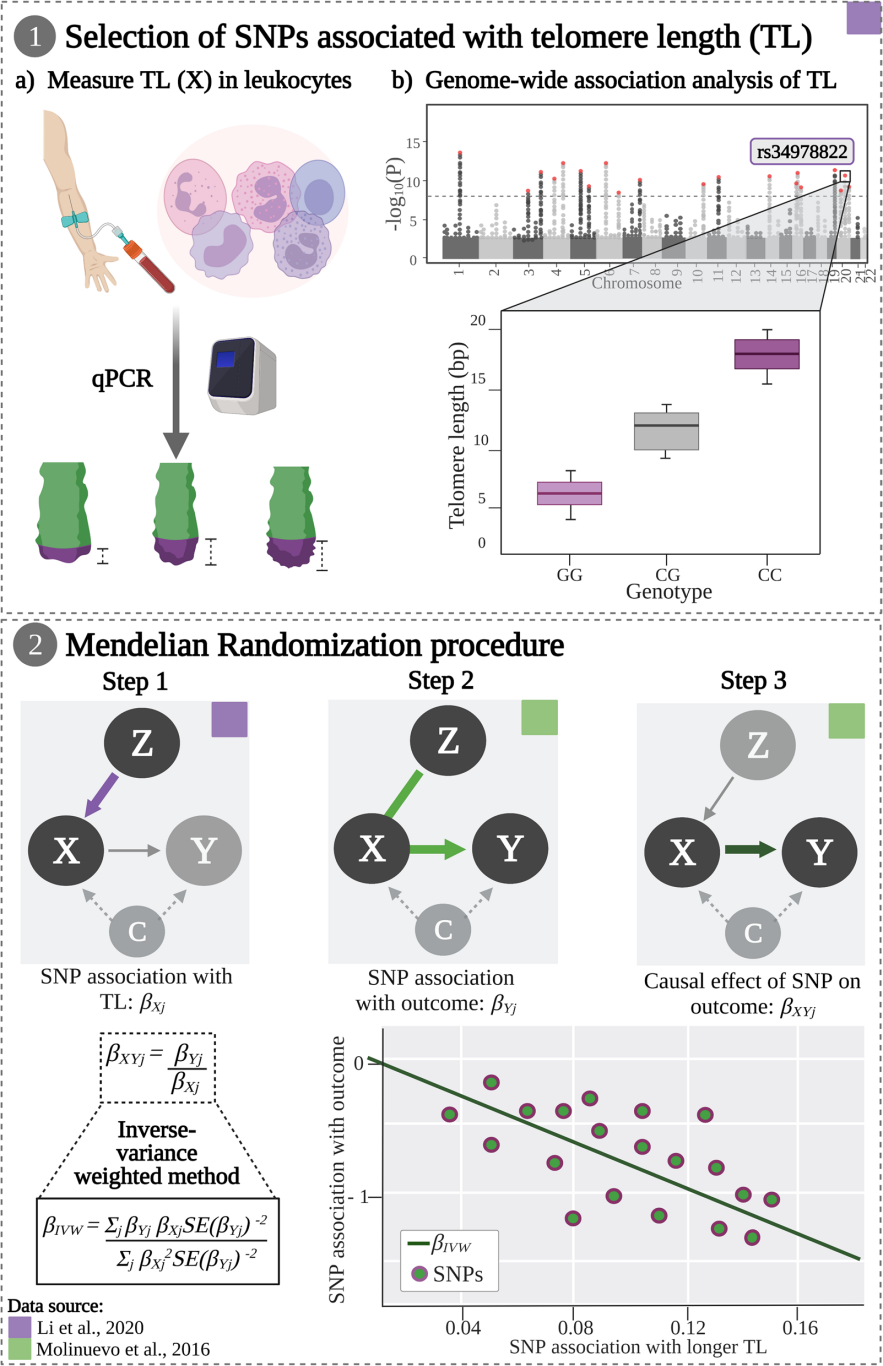
Workflow diagram of the Mendelian randomization method. (1) Selection of single nucleotide polymorphisms (SNPs) associated with telomere length (TL). (a) TL is measured in peripheral blood leukocytes through qPCR. (b) Genome-wide association analysis (GWAS) is performed to identify genome-wide hits (Z) associated with mean TL (X) through linear regression models. (2) Mendelian randomization procedure is performed using summary statistics from the GWAS and individual-level data including genotypic information and the outcomes of interest (Y). ${\beta}_{X_j}$, SNP association with TL from the GWAS summary statistics; ${\beta}_{Y_j}$, SNP association with each outcome is calculated through linear regression models using individual-level data for genotypes and outcomes; ${\beta}_{X{Y}_j}$, causal effect of each SNP on one given outcome; *β*_*IVW*_, inverse-variance weighted (IVW) method is applied to obtain a global estimate representing the causal effect of genetically predicted TL on one given outcome while avoiding confounding (C)

Alleles were harmonized to take as reference the effect allele associated with longer TL. In this study, genetic associations with longer TL were obtained from the summary statistics of a previously computed meta-GWAS [47]. Furthermore, genetic associations with the outcomes (i.e., AD endophenotypes) were assessed in our sample by linear regression models including age, sex, years of education, and *APOE*-ε4 status as covariates (see Additional file 2: Supplementary Tables 1-15; Fig. 1). We used inverse-variance weighted (IVW) [62], maximum likelihood [63], weighted median [64], and weighted mode [65] methods with summarized data to estimate the causal effect of genetically predicted longer TL on the outcomes of the study in a two-sample MR design.

Leave-one-SNP-out and Cochran *Q* statistic [66] were used as ad hoc sensitivity analyses for evaluating the robustness of significant results. MR-Pleiotropy RESidual Sum and Outlier (MR-PRESSO) was also used to identify horizontal pleiotropic outliers [67]. MR-Egger regression intercept test was used as ad hoc sensitivity analysis for evaluating directional pleiotropy [68]. Effect sizes were reported in the SD change (*β*_*IVW*_) per copy of the allele associated with longer TL. Causal associations with *p*-values below 0.05 were considered to be nominal significant. False discovery rate (FDR) corrections for multiple comparisons were performed at a significant level of 0.05 [69, 70]. *p*-value correction was performed within AD endophenotypes and separately in the whole sample and stratified analyses. Models were assessed in the entire sample, as well as stratified by *APOE*-ε4 status and PRS-AD. All analyses were conducted under R software, version 4.1.0 [71]. MR analyses were performed using the *MendelianRandomization* package [72]. Description of study participants was performed using the *compareGroups* R package [49].

## Results

### Descriptive of study participants

Characteristics of ALFA study participants included in our study can be found in Additional file 3: Supplementary Tables 1-3. The final sample size was determined by the number of participants with available genotype information (*N* = 2233) and the number of participants with available information for each one of the main outcomes included in the study, that is, cognitive performance (*N* = 2233), neuroimaging signatures (*N* = 1134), and CSF biomarkers (*N* = 304).

### Mendelian randomization results

#### Cognition outcomes

No statistically significant associations were identified between genetically predicted longer TL and any of the cognitive outcomes in the entire sample, nor in stratified analyses by *APOE*-ɛ4 status or by the genetic predisposition to AD based on high/low categories of PRS-AD (Additional file 4: Supplementary Tables 1-5).

#### Neuroimaging outcomes

**Fig. 2:**
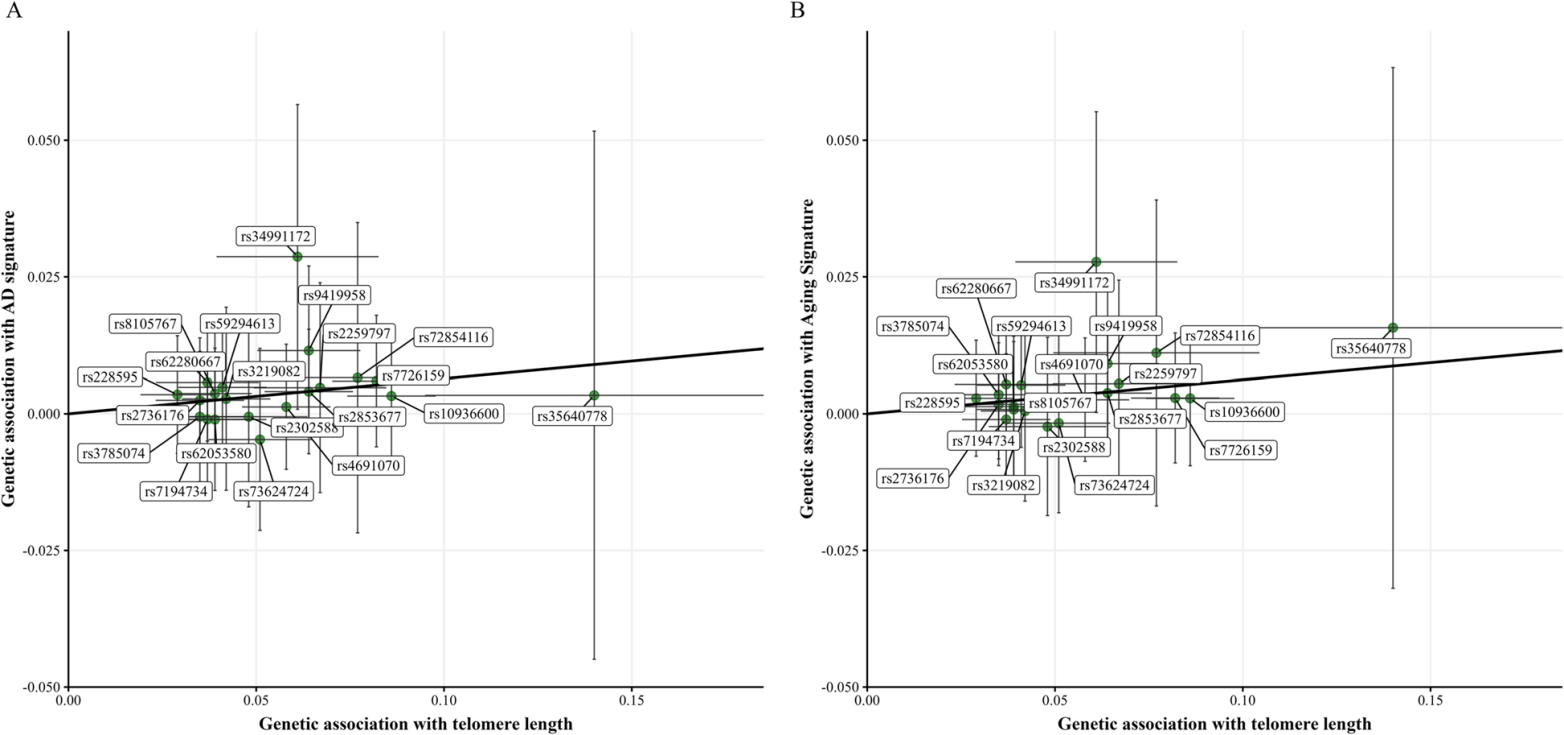
Scatter plot representing the significant causal effects of genetically predicted longer telomere length (TL) on neuroimaging outcomes among individuals at high genetic predisposition to AD. **A** Causal effect of genetically predicted TL on AD signature. **B** Causal effect of genetically predicted TL on aging signature. Each dot represents the genetic association of one given SNP with TL in the *X*-axis and the genetic association of this SNP with either AD or aging signature in the *Y-*axis. The slope of the line represents the inverse-variance weighted causal estimate

IVW estimates revealed statistically significant associations between genetically predicted longer TL and greater cortical thickness for both AD and aging signatures only in individuals at high genetic predisposition to AD [AD signature: *β*_*IVW*_ = 0.064, SE = 0.029, *q*-value = 0.032; aging signature: *β*_*IVW*_ = 0.062, SE = 0.029, *q*-value = 0.032] (Fig. 2A, B; Table 1). Maximum likelihood, weighted median, and weighted mode methods suggested similar patterns of effect on cortical thickness. The MR-Egger intercept test did not provide evidence for directional pleiotropy [AD signature: *p*-value = 0.998; aging signature: *p*-value = 0.939]. Furthermore, according to both Cochran *Q* and MR-PRESSO, there was no evidence of increased heterogeneity (see Table 1). IVW leave-one-out analyses showed MR estimates did not fluctuate after sequentially removing each of the 20 SNPs used as instrumental variables (Additional file 5: Supplementary Figs. 1-2). No other statistically significant associations were found on individuals at low genetic predisposition to AD, as well as in the entire sample, or in stratified analyses by *APOE*-ɛ4 status (Additional file 4: Supplementary Tables 1-5).

**Table 1:** Statistically significant results reflecting the effect of genetically predicted longer telomere length on neuroimaging outcomes

	**High genetic predisposition to AD (*N*= 295)**							
	**AD signature**				**Aging signature**			
**Causal inference methods**	***β***	**SE**	***p*-value**	*q*-value*	***β***	**SE**	***p*-value**	*q*-value*
*Inverse-variance weighted*	0.064	0.029	2.89E−02	3.18E−02	0.062	0.029	3.18E−02	3.18E−02
*Maximum likelihood*	0.064	0.029	2.88E−02	3.15E−02	0.062	0.029	3.15E−02	3.15E−02
*Weighted median*	0.064	0.039	9.91E−02	1.98E−01	0.042	0.038	2.65E−01	2.65E−01
*Weighted mode*	0.058	0.048	2.31E−01	3.68E−01	0.042	0.047	3.68E−01	3.68E−01
**Sensitivity methods**	***p*-value**				***p*-value**			
*Cochran Q, heterogeneity test*	0.995				0.998			
*MR-PRESSO, global test*	0.997				0.998			
*MR-Egger, intercept test*	0.998				0.939			

*AD* Alzheimer’s disease, *PRESSO* Pleiotropy RESidual Sum and Outlier, *SE* standard error

*q-value refers to the false discovery rate-adjusted *p*-value

#### Core AD and neurodegeneration CSF biomarkers

**Fig. 3:**
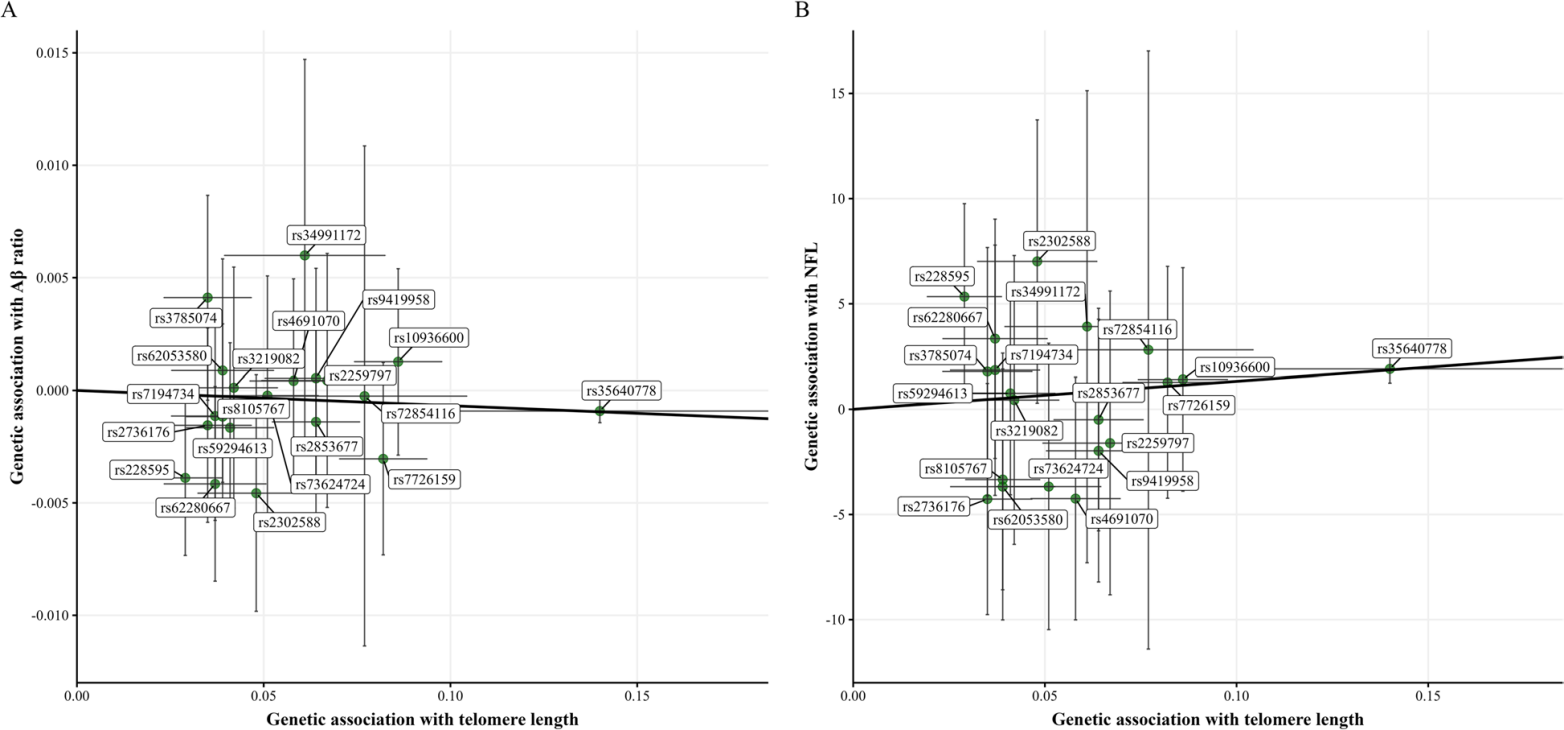
Scatter plot representing the significant causal effects of genetically predicted longer telomere length (TL) on CSF biomarkers in *APOE*-ε4 non-carriers. **A** Causal effect of genetically predicted TL on Aβ ratio. **B** Causal effect of genetically predicted TL on CSF NfL. Each dot represents the genetic association of one given SNP with TL in the *X*-axis and the genetic association of this SNP with CSF Aβ ratio in the *Y*-axis. The slope of the line represents the inverse-variance weighted causal estimate

Statistically significant associations were found between genetically predicted longer TL and lower levels of CSF Aβ ratio [*β*_*IVW*_ = −0.007, SE = 0.002, q-value = 0.001] as well as higher levels of CSF NfL [*β*_*IVW*_ = 13.267, SE = 2.604, *p*-value < 0.0001] only in *APOE*-ɛ4 non-carriers (Fig. 3A, B; Table 2). Robust MR methods suggested significantly analogous patterns of effect on both CSF Aβ ratio and NfL. There was no evidence of unbalanced directional pleiotropy according to the MR-Egger intercept test [CSF Aβ ratio: *p*-value = 0.170 ; CSF NfL: *p*-value = 0.742]. Cochran *Q* and MR-PRESSO also indicated the paucity of heterogeneity in MR estimates (see Table 2). Nevertheless, the leave-one-out analysis showed IVW estimates lost significance when excluding rs35640778 from both MR analyses (Additional file 5: Supplementary Figs. 3-4). Among *APOE*-ɛ4 carriers, MR-PRESSO indicated the presence of increased heterogeneity due to the inclusion of the outlier rs228595 in the analyses (Additional file 4: Supplementary Table 2). However, the MR estimate remained null after correction of pleiotropy via outlier removal.

**Table 2:** Statistically significant results reflecting the effect of genetically predicted longer telomere length on CSF biomarkers

	***APOE*-ɛ4 non-carriers (*N*= 157)**								**High genetic AD risk (*N*= 106)**			
	**Aβ ratio**				**NfL**				**p-tau**			
**Causal inference methods**	***β***	**SE**	***p*-value**	*q*-value*	***β***	**SE**	***p*-value**	*q*-value*	***β***	**SE**	***p*-value**	*q*-value*
*Inverse-variance weighted*	−0.007	0.002	3.26E−04	6.52E−04	13.267	2.604	3.50E−07	1.40E−06	−9.186	4.534	4.28E−02	1.48E−01
*Maximum likelihood*	−0.007	0.002	2.19E−03	4.37E−03	13.044	3.312	8.22E−05	3.29E−04	−8.932	4.563	5.03E−02	1.68E−01
*Weighted median*	−0.007	0.002	4.41E−03	8.83E−03	13.627	3.568	1.34E−04	5.37E−04	−8.571	6.224	1.69E−01	3.07E−01
*Weighted mode*	−0.007	0.002	2.62E−03	5.24E−03	13.327	3.259	4.33E−05	1.73E−04	−7.867	7.917	3.20E−01	5.38E−01
**Sensitivity methods**	***p-value***				***p-value***				***p-value***			
*Cochran Q, heterogeneity test*	0.412				0.254				0.921			
*MR-PRESSO, global test*	0.602				0.505				0.911			
*MR-Egger, intercept test*	0.170				0.742				0.543			

*Aβ* amyloid-β, *AD* Alzheimer’s disease, *CSF* cerebrospinal fluid, *NfL* neurofilament light, *PRESSO* Pleiotropy RESidual Sum and Outlier, *SE* standard error

*q-value refers to the false discovery rate-adjusted *p*-value

**Fig. 4:**
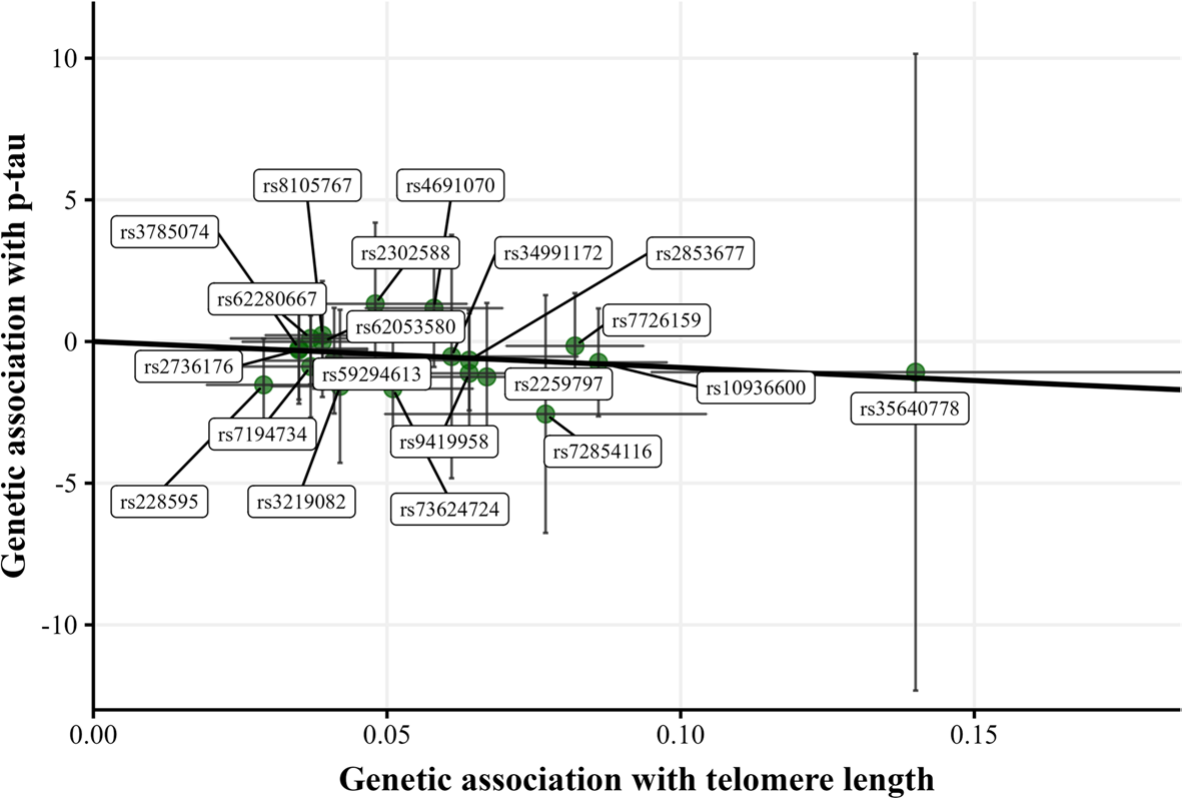
Scatter plot representing the significant causal effect of genetically predicted longer TL on CSF p-tau among individuals at high genetic predisposition to AD. Each dot represents the genetic association of one given SNP with TL in the *X*-axis and the genetic association of this SNP with an aging signature in the *Y*-axis. The slope of the line represents the inverse-variance weighted causal estimate

In stratified analysis by PRS-AD, a nominal statistically significant association was found between SNPs predicting longer TL and lower levels of CSF p-tau only in individuals classified as high genetic predisposition to AD [*β*_*IVW*_ = −9.186, SE = 4.534, *p*-value = 0.043] (Fig. 4; Table 2). No violations of MR assumptions were found according to the MR-Egger intercept test, Cochran *Q*, and MR-PRESSO. Furthermore, MR showed similar estimates when sequentially leaving out genetic variants from the analyses (Additional file 5: Supplementary Fig. 5). No other statistically significant associations were identified in the entire sample in relation to genetically predicted TL and CSF biomarkers.

## Discussion

In this study, we explored the potential causal role of genetically predicted longer TL on AD endophenotypes measured as cognitive performance, brain structure (i.e., AD signature and aging signature), and CSF biomarkers of AD and neurodegeneration, in cognitively unimpaired individuals from the ALFA study. We performed stratified analysis by *APOE*-ɛ4 status and the estimated genetic predisposition of developing AD (i.e., PRS-AD). We observed statistically significant associations between genetically predicted longer TL, lower levels of CSF Aβ ratio, and higher levels of CSF NfL only in *APOE*-ɛ4 non-carriers. Additionally, stratified analysis by the PRS-AD revealed statistically significant associations between SNPs predicting longer TL, greater cortical thickness in AD and aging signatures and lower levels of p-tau only among individuals at high genetic predisposition of developing the disease.

### Genetically predicted TL and cognitive performance

We did not observe any statistically significant association between genetically predicted longer TL and cognition outcomes. Previous literature showed inconsistent findings regarding TL effect on cognitive performance. Similar to our approach, Hägg et al. [31] reported an association between genetically predicted longer TL and better scores on EF, but not for global cognition or EM, in a large cohort of cognitively healthy individuals (*N*= 17, 052). Most recently, Demanelis et al. [32] found genetically predicted TL to be associated with several aging-related traits but not cognitive function among participants of the UK Biobank (*N* = 337, 522).

Regarding observational studies, Yaffe et al. [73] reported an association between longer TL and better performance at baseline on the EF domain among community-dwelling elders. In contrast, longer TL at baseline has also been associated with a decline in EF only among cognitively healthy *APOE*-ε4 carriers [22]. Albeit, Takata et al. [74] did not find any association between TL and cognitive decline. In the Rotterdam study (*N* = 1961) a U-shaped association between TL and risk of AD was found, meaning that both, extremely shorter TL as well as extremely longer TL, were associated with an increased risk of AD, with stronger associations seen for *APOE*-ε4 carriers [75]. Alternatively, shorter TL has been shown to predict AD incidence only among *APOE*-ε4 non-carriers [76].

In the present study, the lack of statistically significant associations may be due to its cross-sectional nature as well as the idiosyncrasies of the ALFA cohort, which is composed of middle-aged (i.e., mean age of 56.3 years) and cognitively unimpaired individuals.

### Genetically predicted TL and brain structure

Our results showed a significant association between genetically predicted longer TL and greater cortical thickness in both the AD and aging brain signatures, only among individuals at high genetic predisposition of AD. To our knowledge, no previous MR analyses have evaluated the causal effect of TL on brain structure, nor in regions primarily affected by AD or normal aging-related atrophy. Longer TL has been previously associated with larger hippocampal volumes in non-demented individuals in observational cross-sectional studies [15, 17]. Furthermore, a population-based study (i.e., Dallas Heart Study) found TL to be associated with total cerebral volume and specific subsegmental regions of the cortex [14].

Mechanisms explaining how TL could affect brain structure during the life-course are not completely understood. TL is known to play a crucial role in brain development with implications in embryonic and adult neurogenesis [77]. However, there is still a scientific debate over the existence of telomere attrition in neurons due to its post-mitotic nature. Even so, telomerase reactivation (i.e., the enzyme that maintains TL in the cell) reversed brain tissue degeneration among aged telomerase-deficient mice [78]. Therefore, inherited longer TL might protect brain structure through multiple mechanisms, either in regions primarily affected by AD-related processes or aging itself.

Stratified analyses did not show significant differences in the association between genetically predicted TL and brain structure in relation to *APOE*-ɛ4, nor PRS-AD subgroups. Similarly, King et al. [14] described that associations between TL and cortical volumes remained significant after adjusting by *APOE*-ε4 status.

### Genetically predicted TL and biomarkers of AD and neurodegeneration

Our results showed significant associations between SNPs predicting longer TL, lower levels of CSF Aβ ratio, and higher levels of CSF NfL in *APOE*-ɛ4 non-carriers. These results suggested an unexpected adverse effect of longer TL on CSF biomarkers related to both core AD pathology and neurodegeneration. However, stratified analysis by PRS-AD suggested a protective effect of inherited longer TL on core AD pathology, with longer TL nominally significant associated with lower levels of p-tau among individuals at high genetic predisposition of AD.

To our knowledge, no previous MR studies have tested the effect of genetically predicted TL on AD biomarkers and observational studies are scarce. TL has been previously associated with AD pathology with implications on cognitive function in participants of the AD Neuroimaging Initiative [22]. Moreover, Flanary et al. [79] showed that senescence of isolated human microglia related to telomere shortening enhanced Aβ accumulation and induced AD progression. However, underlying biological mechanisms explaining the association are not fully understood yet.

Given the complexity of microglia homeostasis [80], we might speculate that unexplored roles of TL on microglia activation could lead to both beneficial or detrimental effects on AD pathology and neurodegeneration processes, which would partially explain the aforementioned disparity of results. However, it remains unclear how *APOE*-ɛ4 carriership and the genetic predisposition of AD would affect this putative association. Interestingly, some of the genetic variants which were used as instrumental variables to estimate genetically predicted telomere length in the present study were primarily related to the risk of cardiovascular disease [81], and they were later found to be causally associated with AD risk [27]. Cardiovascular risk factors comprise important targets for preventing AD especially during midlife [24]. Indeed, midlife vascular risk factors have been previously associated with elevated amyloid burden [82], tau pathology, and cognition [83], supporting their role on the etiology of AD. Thus, the TL effect on cardiovascular health outcome could be mediating or contributing to the causal associations described in our cohort. Nevertheless, further studies with larger sample sizes are required to better understand these results since a variety of undisclosed pathological mechanisms could be responsible for this complex interplay.

### Limitations and strengths

Our study is not free of limitations. Potential limitations could be due to MR assumptions. For instance, IVW estimates could be biased in the presence of pleiotropy. To ensure estimates were reliable, sensitivity analyses through the MR-Egger method were performed. Nevertheless, we cannot completely ensure whether other unexplored factors are confounding these effects [62]. In addition, our sample size is small when compared to other MR studies, which can result in a relative loss of significance when exploring multiple MR methods with different assumptions. However, we ensured the inclusion of strong instruments by applying a two-sample MR approach in which the estimates of the genetic associations with the exposure of interest (i.e., longer TL) were calculated in a previous genome-wide meta-analysis of 78,592 individuals.

It is noteworthy that our cohort is rather selected and composed of middle-aged cognitively unimpaired individuals. This could entail a recruitment bias, particularly in individuals at high genetic predisposition of AD. Individuals at high genetic predisposition might have already shown signs of cognitive decline at recruitment, which was an exclusion criterion of the present study. Furthermore, this also implies that our participants showed a low prevalence of other common comorbidities and that our results might not be generalizable to individuals at more advanced stages in the AD *continuum*. Indeed, given its cross-sectional nature, endophenotypes included in the present study might reflect a transient stage within the AD pathological process. Finally, those associations that did not survive FDR multiple comparisons correction should be interpreted with caution.

Despite the aforementioned limitations, the strengths of our study include the robust and well-characterized cohort of cognitively unimpaired middle-aged individuals. This included the extensive characterization of cognitive outcomes, the use of high-resolution brain scans, the quantification of AD biomarkers and neurodegeneration biomarkers in CSF, and the genome-wide genotyping. Another important strength is also the higher proportion of *APOE*-ε4 carriers in the ALFA participants compared to the general population (19% vs. 14% in the general population; *p* < 0.001) [84] and the available PRS of AD. Although carriership of the *APOE*-ɛ4 allele is the largest genetic risk factor of AD, PRS of AD has been also associated with cognitive decline [85], hippocampal function [86], and core AD CSF biomarkers [87]. In this sense, this study also has evidenced the benefits of using PRS-AD to characterize individuals at high genetic predisposition of AD (besides *APOE*) in population-based cohort studies and elucidate specific biological patterns associated with telomere length homeostasis.

To our knowledge, no prior MR studies have explored the potential causal association between TL and these AD endophenotypes (i.e., cognitive performance, AD and aging brain signatures based on brain structure and biomarkers of AD, and neurodegeneration in CSF) in a similar cohort of cognitively unimpaired individuals at increased risk of developing AD. Thus, our findings should be replicated in larger cohorts including AD patients at more advanced stages of the disease. Similarly, the follow-up of ALFA participants and further observational analyses are required to further understand such observations.

## Conclusions

In conclusion, the results of this cross-sectional study show that genetically predicted longer TL may protect brain structure in cognitively unimpaired individuals. In addition, *APOE* alleles and the genetic predisposition to AD could modulate TL effects on CSF biomarkers of AD and neurodegeneration. This result opens new avenues to study the role of telomeres on AD progression.

## Supplementary Information

**Additional file 1: Supplementary Table 1.** Characteristics of Single Nucleotide Polymorphisms (SNPs) associated with longer telomere length. The effect allele refers to the allele that is associated with longer telomere length. Chromosomal position of the SNPs (genome assembly GRCh37 (hg19)) according to the public archive for genetic variation within and across different species developed and hosted by the National Center for Biotechnology Information (NCBI) in collaboration with the National Human Genome Research Institute (NHGRI) (dbSNP).

**Additional file 2: Supplementary Table 1.** Linear regression estimates for cognition outcomes in the entire sample. All models are adjusted for covariates: age, sex, education, and *APOE* status. **Supplementary Table 2.** Linear regression estimates for neuroimaging outcomes (*i.e.*, Alzheimer’s disease and aging signatures) outcome in the entire sample. All models are adjusted for covariates: age, sex, education, and *APOE* status. **Supplementary Table 3.** Linear regression estimates for CSF biomarkers outcomes in the entire sample. All models are adjusted for covariates: age, sex, education, and *APOE* status. **Supplementary Table 4.** Linear regression estimates for cognition outcomes in *APOE*-ɛ4 carriers. All models are adjusted for covariates: age, sex, and education. **Supplementary Table 5.** Linear regression estimates for neuroimaging outcomes (*i.e.*, Alzheimer’s disease and aging signatures) in *APOE*-ɛ4 carriers. All models are adjusted for covariates: age, sex, and education. **Supplementary Table 6.** Linear regression estimates for CSF biomarkers outcomes in *APOE*-ɛ4 carriers. All models are adjusted for covariates: age, sex, and education. **Supplementary Table 7.** Linear regression estimates for cognition outcomes in *APOE*-ɛ4 non-carriers. All models are adjusted for covariates: age, sex, and education. **Supplementary Table 8.** Linear regression estimates for neuroimaging outcomes (*i.e.*, Alzheimer’s disease and aging signatures) in *APOE*-ɛ4 non-carriers. All models are adjusted for covariates: age, sex, and education. **Supplementary Table 9.** Linear regression estimates for CSF biomarkers outcomes in *APOE*-ɛ4 carriers. All models are adjusted for covariates: age, sex, and education. **Supplementary Table 10.** Linear regression estimates for cognition outcomes among individuals at high genetic predisposition to AD. All models are adjusted for covariates: age, sex, and education. **Supplementary Table 11.** Linear regression estimates for neuroimaging outcomes (*i.e.*, Alzheimer’s disease and aging signatures) among individuals at high genetic predisposition to AD. All models are adjusted for covariates: age, sex, and education. **Supplementary Table 12.** Linear regression estimates for CSF biomarkers outcomes (*i.e.*, Alzheimer’s disease and aging signatures) among individuals at high genetic predisposition to AD. All models are adjusted for covariates: age, sex, and education. **Supplementary Table 13.** Linear regression estimates for cognition outcomes among individuals at low genetic predisposition to AD. All models are adjusted for covariates: age, sex, and education. **Supplementary Table 14.** Linear regression estimates for neuroimaging outcomes (*i.e.*, Alzheimer’s disease and aging signatures) among individuals at low genetic predisposition to AD. All models are adjusted for covariates: age, sex, and education. **Supplementary Table 15.** Linear regression estimates for CSF biomarkers outcomes among individuals at low genetic predisposition to AD. All models are adjusted for covariates: age, sex, and education.

**Additional file 3: Supplementary Table 1.** Characteristics of the study participants with information for cognition outcomes. Mean and SD are shown for continuous variables. **Supplementary Table 2.** Characteristics of the study participants with information for neuroimaging outcomes. Mean and SD are shown for continuous variables. **Supplementary Table 3.** Characteristics of the study participants with information for CSF biomarkers. Mean and SD are shown for continuous variables.

**Additional file 4: Supplementary Table 1.** Results of the effect of genetically predicted longer telomere length on AD endophenotypes in the entire sample. **Supplementary Table 2.** Results of the effect of genetically predicted longer telomere length on AD endophenotypes among *APOE*-ɛ4 carriers. Supplementary Table 3. Results of the effect of genetically predicted longer telomere length on AD endophenotypes among *APOE*-ɛ4 non-carriers. **Supplementary Table 4.** Results of the effect of genetically predicted longer telomere length on AD endophenotypes among individuals at high genetic predisposition to Alzheimer's disease. **Supplementary Table 5.** Results of the effect of genetically predicted longer telomere length on AD endophenotypes among individuals at low genetic predisposition to Alzheimer's disease.

**Additional file 5: Supplementary Figure 1.** Leave-one-out permutation analysis plot for AD signature among individuals at high genetic predisposition to AD obtained by leaving out the SNP indicated and repeating the Inverse-Variance Weighted method with the rest of the instrumental variables. **Supplementary Figure 2.** Leave-one-out permutation analysis plot for Aging signature among individuals at high genetic predisposition to AD, obtained by leaving out the SNP indicated and repeating the Inverse-Variance Weighted method with the rest of the instrumental variables. **Supplementary Figure 3.** Leave-one-out permutation analysis plot for Aβ ratio among *APOE*-ɛ4 non-carriers obtained by leaving out the SNP indicated and repeating the Inverse-Variance Weighted method with the rest of the instrumental variables. **Supplementary Figure 4.** Leave-one-out permutation analysis plot for NfL among *APOE*-ɛ4 non-carriers obtained by leaving out the SNP indicated and repeating the Inverse-Variance Weighted method with the rest of the instrumental variables. **Supplementary Figure 5.** Leave-one-out permutation analysis plot for p-tau among individuals at high genetic predisposition to AD, obtained by leaving out the SNP indicated and repeating the Inverse-Variance Weighted method with the rest of the instrumental variables.

## Data Availability

The data that support the findings of this study are available from the corresponding authors (NVT, MCB), upon reasonable request.

## Abbreviations

Aβ: Amyloid-β
AD: Alzheimer’s disease
APOE: Apolipoprotein E
CSF: Cerebrospinal fluid
DNA: Deoxyribonucleic acid
EF: Executive function
EM: Episodic memory
GWAS: Genome-wide association study
IVW: Inverse-variance weighted
MBT: Memory Binding Test
MCI: Mild cognitive impairment
MR: Mendelian randomization
MRI: Magnetic resonance imaging
NfL: Neurofilament light
PACC: Preclinical Alzheimer Cognitive Composite
PRESSO: Pleiotropy RESidual Sum and Outlier
PRS: Polygenic risk score
QC: Quality control
SE: Standard error
SD: Standard deviation
SNP: Single nucleotide polymorphism
TL: Telomere length
WAIS: Wechsler Adult Intelligence Scale
