# Supplementary figures and images for: Genetically predicted telomere length and Alzheimer’s disease endophenotypes: a Mendelian randomization study
Source: Alzheimers Res Ther. 2022 Nov 7;14:167. doi: 10.1186/s13195-022-01101-9 (PMC9641781; doi:10.1186/s13195-022-01101-9)

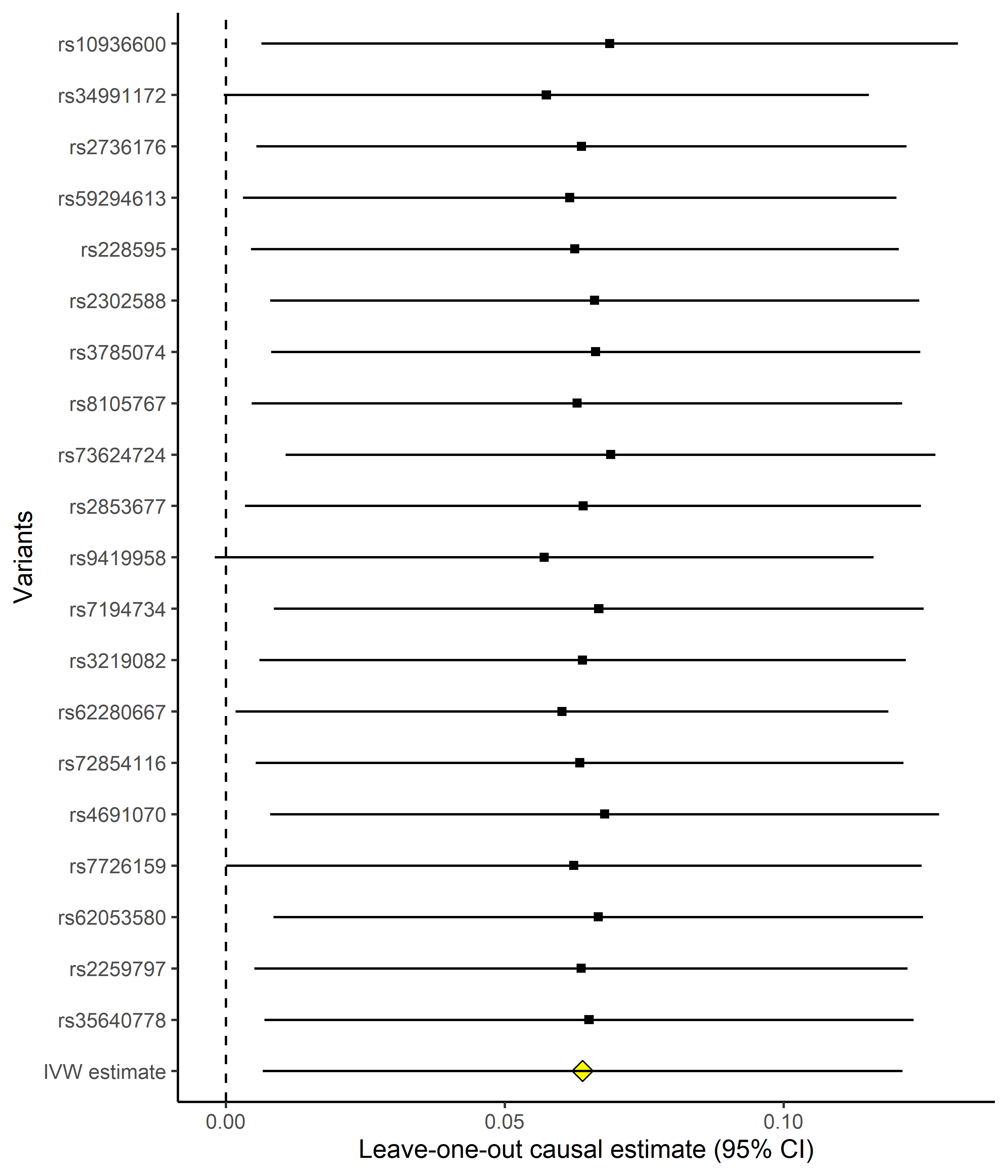

Supplement: Supplementary file 5 — Additional file 5: Supplementary Figure 1. Leave-one-out permutation analysis plot for AD signature among individuals at high genetic predisposition to AD obtained by leaving out the SNP indicated and repeating the Inverse-Variance Weighted method with the rest of the instrumental variables. Supplementary Figure 2. Leave-one-out permutation analysis plot for Aging signature among individuals at high genetic predisposition to AD, obtained by leaving out the SNP indicated and repeating the Inverse-Variance Weighted method with the rest of the instrumental variables. Supplementary Figure 3. Leave-one-out permutation analysis plot for Aβ ratio among APOE-ɛ4 non-carriers obtained by leaving out the SNP indicated and repeating the Inverse-Variance Weighted method with the rest of the instrumental variables. Supplementary Figure 4. Leave-one-out permutation analysis plot for NfL among APOE-ɛ4 non-carriers obtained by leaving out the SNP indicated and repeating the Inverse-Variance Weighted method with the rest of the instrumental variables. Supplementary Figure 5. Leave-one-out permutation analysis plot for p-tau among individuals at high genetic predisposition to AD, obtained by leaving out the SNP indicated and repeating the Inverse-Variance Weighted method with the rest of the instrumental variables. [file 13195_2022_1101_MOESM5_ESM.zip › SupplementaryFigure1.tiff]

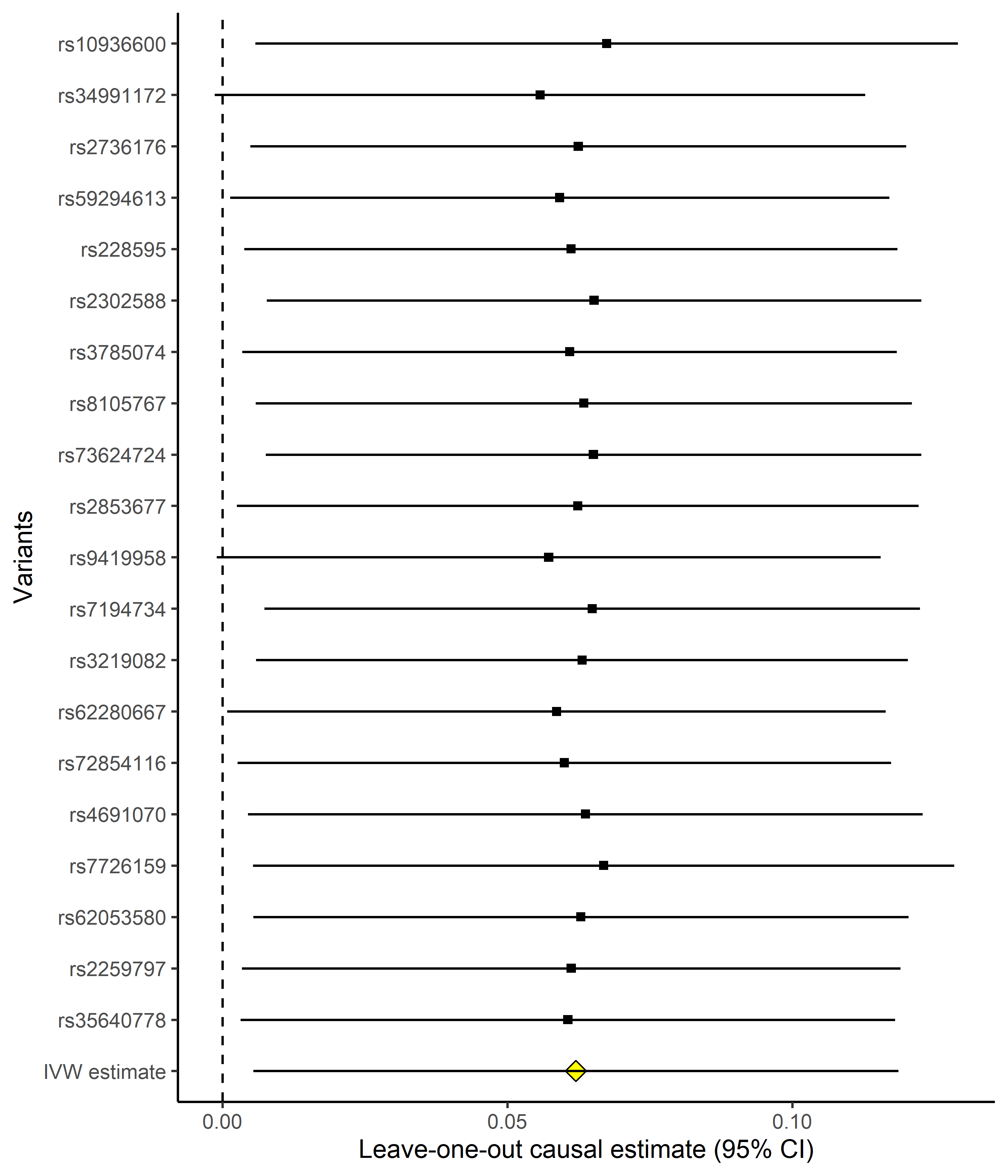

Supplement: Supplementary file 5 — Additional file 5: Supplementary Figure 1. Leave-one-out permutation analysis plot for AD signature among individuals at high genetic predisposition to AD obtained by leaving out the SNP indicated and repeating the Inverse-Variance Weighted method with the rest of the instrumental variables. Supplementary Figure 2. Leave-one-out permutation analysis plot for Aging signature among individuals at high genetic predisposition to AD, obtained by leaving out the SNP indicated and repeating the Inverse-Variance Weighted method with the rest of the instrumental variables. Supplementary Figure 3. Leave-one-out permutation analysis plot for Aβ ratio among APOE-ɛ4 non-carriers obtained by leaving out the SNP indicated and repeating the Inverse-Variance Weighted method with the rest of the instrumental variables. Supplementary Figure 4. Leave-one-out permutation analysis plot for NfL among APOE-ɛ4 non-carriers obtained by leaving out the SNP indicated and repeating the Inverse-Variance Weighted method with the rest of the instrumental variables. Supplementary Figure 5. Leave-one-out permutation analysis plot for p-tau among individuals at high genetic predisposition to AD, obtained by leaving out the SNP indicated and repeating the Inverse-Variance Weighted method with the rest of the instrumental variables. [file 13195_2022_1101_MOESM5_ESM.zip › SupplementaryFigure2.tiff]

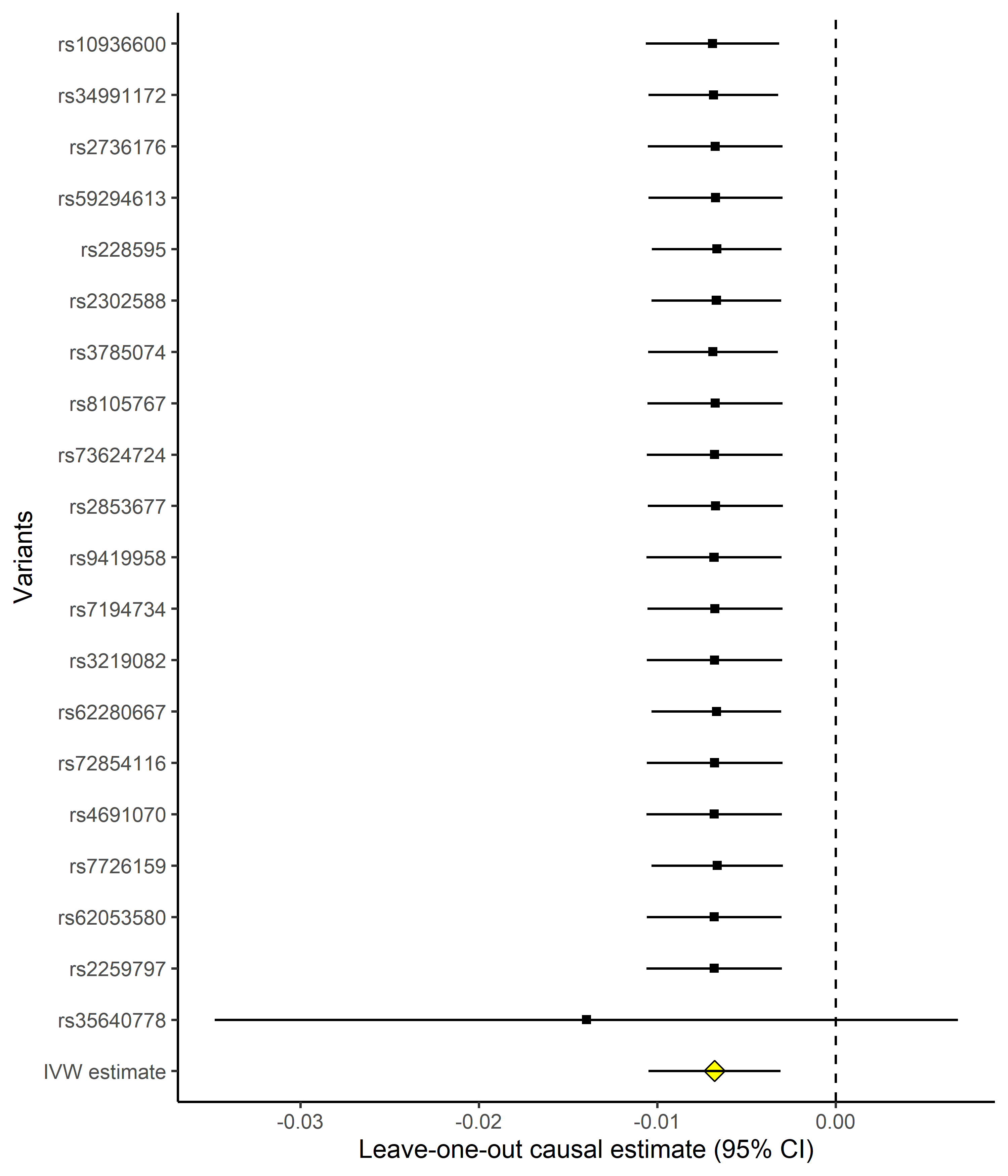

Supplement: Supplementary file 5 — Additional file 5: Supplementary Figure 1. Leave-one-out permutation analysis plot for AD signature among individuals at high genetic predisposition to AD obtained by leaving out the SNP indicated and repeating the Inverse-Variance Weighted method with the rest of the instrumental variables. Supplementary Figure 2. Leave-one-out permutation analysis plot for Aging signature among individuals at high genetic predisposition to AD, obtained by leaving out the SNP indicated and repeating the Inverse-Variance Weighted method with the rest of the instrumental variables. Supplementary Figure 3. Leave-one-out permutation analysis plot for Aβ ratio among APOE-ɛ4 non-carriers obtained by leaving out the SNP indicated and repeating the Inverse-Variance Weighted method with the rest of the instrumental variables. Supplementary Figure 4. Leave-one-out permutation analysis plot for NfL among APOE-ɛ4 non-carriers obtained by leaving out the SNP indicated and repeating the Inverse-Variance Weighted method with the rest of the instrumental variables. Supplementary Figure 5. Leave-one-out permutation analysis plot for p-tau among individuals at high genetic predisposition to AD, obtained by leaving out the SNP indicated and repeating the Inverse-Variance Weighted method with the rest of the instrumental variables. [file 13195_2022_1101_MOESM5_ESM.zip › SupplementaryFigure3.tiff]

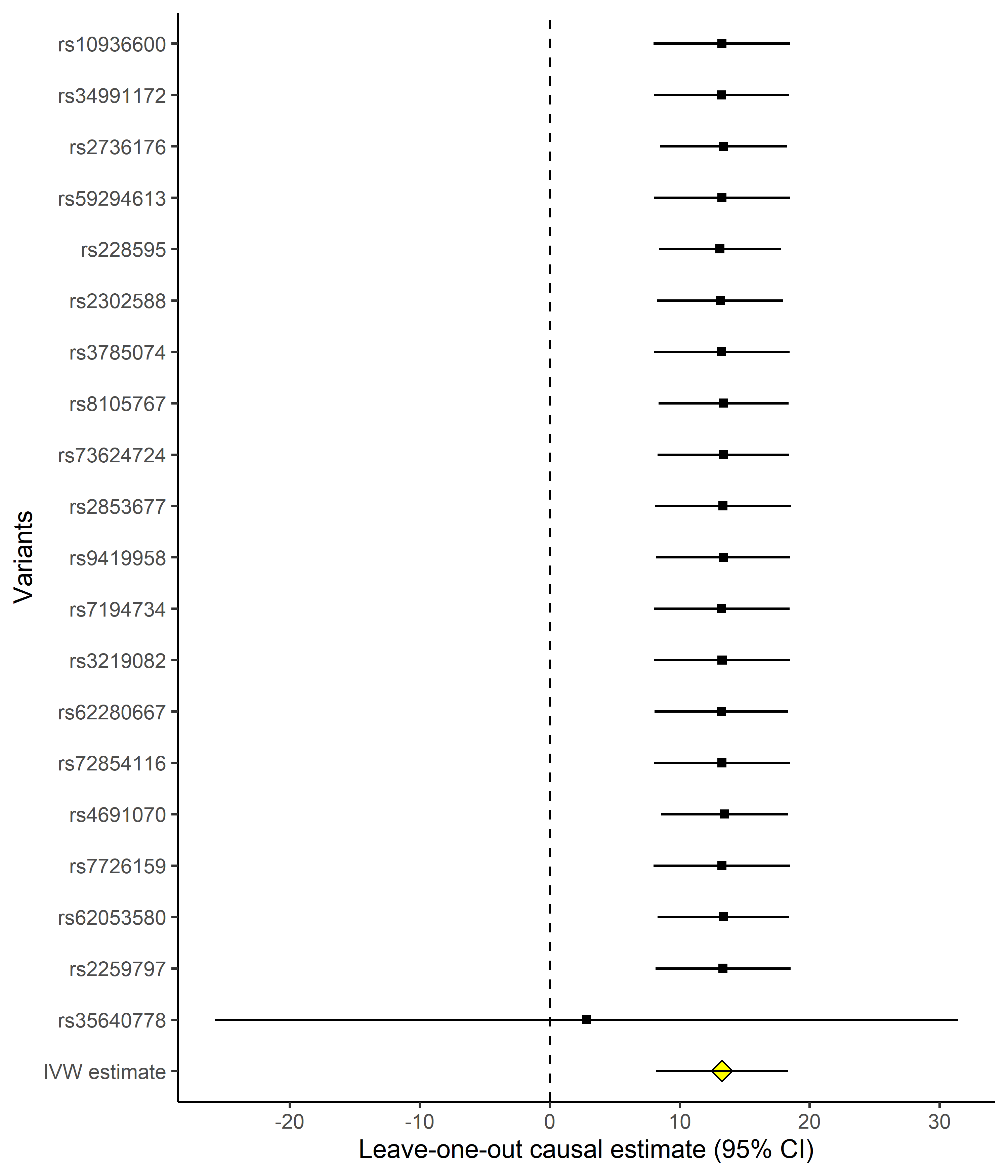

Supplement: Supplementary file 5 — Additional file 5: Supplementary Figure 1. Leave-one-out permutation analysis plot for AD signature among individuals at high genetic predisposition to AD obtained by leaving out the SNP indicated and repeating the Inverse-Variance Weighted method with the rest of the instrumental variables. Supplementary Figure 2. Leave-one-out permutation analysis plot for Aging signature among individuals at high genetic predisposition to AD, obtained by leaving out the SNP indicated and repeating the Inverse-Variance Weighted method with the rest of the instrumental variables. Supplementary Figure 3. Leave-one-out permutation analysis plot for Aβ ratio among APOE-ɛ4 non-carriers obtained by leaving out the SNP indicated and repeating the Inverse-Variance Weighted method with the rest of the instrumental variables. Supplementary Figure 4. Leave-one-out permutation analysis plot for NfL among APOE-ɛ4 non-carriers obtained by leaving out the SNP indicated and repeating the Inverse-Variance Weighted method with the rest of the instrumental variables. Supplementary Figure 5. Leave-one-out permutation analysis plot for p-tau among individuals at high genetic predisposition to AD, obtained by leaving out the SNP indicated and repeating the Inverse-Variance Weighted method with the rest of the instrumental variables. [file 13195_2022_1101_MOESM5_ESM.zip › SupplementaryFigure4.tiff]

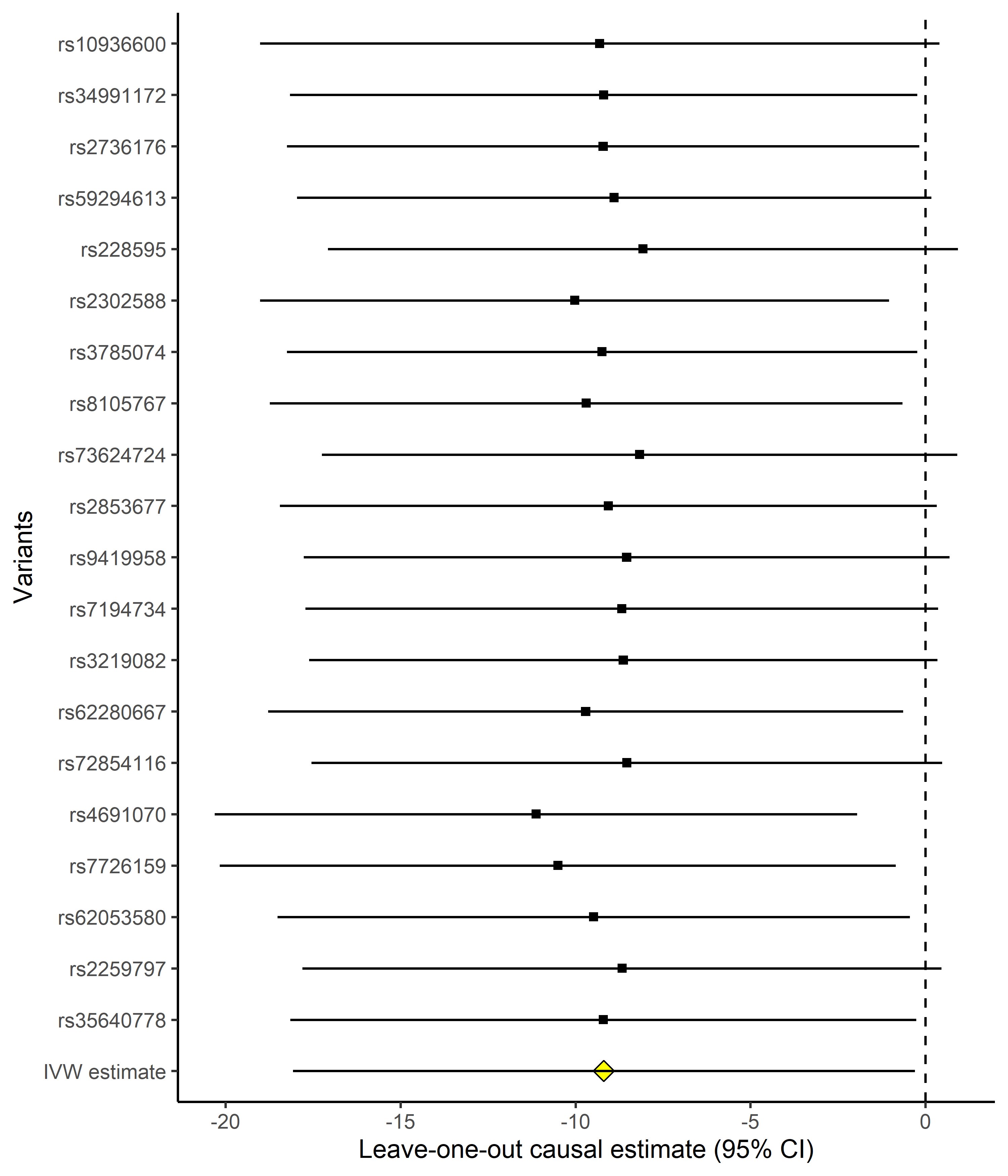

Supplement: Supplementary file 5 — Additional file 5: Supplementary Figure 1. Leave-one-out permutation analysis plot for AD signature among individuals at high genetic predisposition to AD obtained by leaving out the SNP indicated and repeating the Inverse-Variance Weighted method with the rest of the instrumental variables. Supplementary Figure 2. Leave-one-out permutation analysis plot for Aging signature among individuals at high genetic predisposition to AD, obtained by leaving out the SNP indicated and repeating the Inverse-Variance Weighted method with the rest of the instrumental variables. Supplementary Figure 3. Leave-one-out permutation analysis plot for Aβ ratio among APOE-ɛ4 non-carriers obtained by leaving out the SNP indicated and repeating the Inverse-Variance Weighted method with the rest of the instrumental variables. Supplementary Figure 4. Leave-one-out permutation analysis plot for NfL among APOE-ɛ4 non-carriers obtained by leaving out the SNP indicated and repeating the Inverse-Variance Weighted method with the rest of the instrumental variables. Supplementary Figure 5. Leave-one-out permutation analysis plot for p-tau among individuals at high genetic predisposition to AD, obtained by leaving out the SNP indicated and repeating the Inverse-Variance Weighted method with the rest of the instrumental variables. [file 13195_2022_1101_MOESM5_ESM.zip › SupplementaryFigure5.tiff]
